# The complete chloroplast genome of a solid type of *Phyllostachys nidularia* (Bambusoideae: Poaceae), a species endemic to China

**DOI:** 10.1080/23802359.2021.1889411

**Published:** 2021-03-18

**Authors:** Zhou Jie, Hu Yaping, Yu Zhaoyan, Li Jiajia, Xu Mingye, Guo Qirong

**Affiliations:** a Co-Innovation Center for Sustainable Forestry in Southern China, Nanjing Forestry University, Nanjing, China; b International Center of Bamboo and Rattan, Beijing, China

**Keywords:** Chloroplast genome, *Phyllostachys nidularia* f. *farcta*, phylogeny, whole genome sequencing data

## Abstract

*Phyllostachys nidularia* (Bambusoideae: Poaceae), widely distributed in the Yangtze River Basin and various provinces (regions) in southern China, is one of the most important small and medium-sized bamboo species used in both bamboo shoots and timber. In the present study, we assembled a complete chloroplast genome of the economically important bamboo form *Phyllostachys nidularia* f. *farcta* H.R.Zhao & A.T.Liu using whole genome sequencing data previously reported. The complete chloroplast (cp) genome is 139,706 bp in length. A total of 129 unique genes were annotated, including 82 protein-coding, 39 tRNA, and eight rRNA genes. Phylogenetic analysis results supported that *P. nidularia* f. *farcta* was closely related to *Phyllostachys reticulata*. This work would help us better understand the evolution of the *Phyllostachys* cp genome.

*Phyllostachys nidularia* (Poaceae, Bambusoideae) is one of the most widely distributed bamboo species in China, with high economic value and ecological benefits. *Phyllostachys nidularia* is closely related to people's life and production, its bamboo shoots are edible, bamboo stalks are important construction, manufacturing, and paper industry materials, and *P. nidularia* is one of the most important economic bamboo species in China (Wu et al. 2006; Zhang et al. 2019). *Phyllostachys nidularia* f. *farcta* (http://www.theplantlist.org/tpl1.1/record/kew-434223) is a form of *P. nidularia*. It traditionally identifies the analyzed specimen as *Phyllostachys nidularia* f. *farcta* in China. Compared with the original form, its culm is solid or nearly solid (McClure 1956). Since *P. nidularia* is increasingly valued by industry, further study of the cp genome will improve our understanding of this species as well as assist in future breeding experiments. Here the complete cp genome sequence of *P. nidularia* f. *farcta* was deciphered.

Samples were collected from shoots growing in Lukou Town, Changsha County, Hunan Province, China (113.11° E, 28.27° N). Total genomic DNA was extracted from bamboo shoot bud tissue and the specimen voucher is deposited in the college of forestry, Nanjing Forestry University (NJFU-2020779). The raw data was uploaded to NCBI with Accession number-SRS6922745. The Hiseq 2500 sequencer was used to obtain 35.4 gb of raw data. After filtering and pruning with fastp (Chen et al. 2018), Novoplasty (v4.0) was used to assemble the high-quality paired end reads into a complete cp genome. Genome annotation was performed with GeSeq (Tillich et al. 2017). The cp genome was annotated using Geneious R8 (v8.0.4) and the result adjusted manually. The annotated chloroplast genome has been submitted to GenBank with accession number LC590826. The phylogenetic analysis included the outgroups *Arundinaria fargesii* and two bamboo species of Indosasa. The phylogeny was inferred using the Maximum likelihood (ML) method. The alignment was achieved with MAFFT (Katoh and Standley 2013), and the phylogenetic tree was constructed using IQ-tree (Minh et al. 2020). The best-fitted model was T92 + G, selected by ModelFinder (Kalyaanamoorthy et al. 2017).

The cp genome was 139,706 bp in size and is quadripartite, consisting of a pair of inverted repeats (IRs, 21,798 bp), a large single copy (LSC, 83,241 bp), and a small single copy (SSC, 12,869 bp). There were 129 unique genes, including 82 protein-coding, 39 tRNA, and eight rRNA genes. Seven protein-coding genes (*rps7*, *rps12*, *rpl2*, *rpl23*, *ndhB*, *ycf68*), eight tRNA genes (*trnA*-*UGC*, *trnH*-*GUG*, *trnI*-*CAU*, *trnI*-*GAU*, *trnL*-*CAA*, *trnN*-*GUU*, *trnR*-*ACG*, *trnV*-*GAC*), and all rRNA genes (4.5S, 5S, 16S, 23S) were located in the IR regions. The GC content of complete cp genome was 38.88%. The GC content of IRs (44.22%) was higher than that of LSC (36.97%) and SSC (33.16%).

The phylogenetic analysis indicated that *P. nidularia* f. *farcta* was closely related to *P. reticulata* (Figure 1). The chloroplast genome of *P. nidularia* f. *farcta* will provide useful genetic information for the further study and conservation of bamboo species. *Phyllostachys* is divided into two groups (Sect. *Phyllostachys* and Sect. *Heteroclada*). We have checked the data several times and found that *Phyllostachys nidularia* and *Phyllostachys reticulata* (MN537808.1) are more similar based on pairwise genetic distance, and do not show closer *Phyllostachys nigra* var. *henonis* of Sect. *Heteroclada* in genetic distance. Previous studies (Zhang et al. 2019) have revealed that some *Phyllostachys* species may have hybridized during their long evolutionary history, which has deepened our understanding of the phylogeny of *Phyllostachys*.

**Figure 1.**
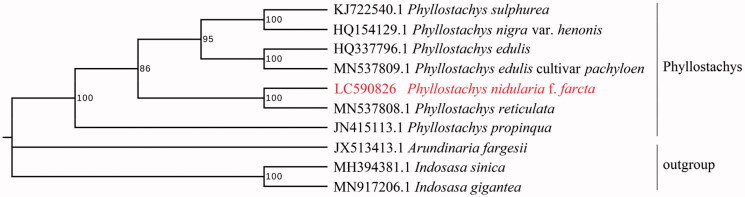
Phylogenetic relationships among *Phyllostachys nidularia* f. *farcta* and 10 complete chloroplast genomes of bamboo species. Bootstrap support values are given at the nodes.

## Data Availability

The genome sequence data that support the findings of this study are openly available in GenBank of NCBI at https://www.ncbi.nlm.nih.gov/ under the accession no. LC590826. The associated BioProject, SRA and Bio-Sample numbers are PRJNA642983, SRS6922745 and SAMN15402429 in NCBI.
